# Determination of glomerular filtration rate “en passant” after high doses of iohexol for computed tomography in intensive care medicine—a proof of concept

**DOI:** 10.3389/fphar.2024.1346343

**Published:** 2024-02-01

**Authors:** Alexander Dejaco, Christoph Dorn, Michael Paal, Michael Gruber, Bernhard M. Graf, Martin G. Kees

**Affiliations:** ^1^ Department of Anesthesia, University Hospital Regensburg, Regensburg, Germany; ^2^ Institute of Pharmacy, University of Regensburg, Regensburg, Germany; ^3^ Institute for Laboratory Medicine, Hospital of the University of Munich (LMU), Munich, Germany

**Keywords:** measured glomerular filtration rate (mGFR), estimated glomerular filtration rate (eGFR), intensive care medicine (ICM), contrast-enhanced computed tomography imaging, “en passant” iohexol clearance, pharmacokinetic studies

## Abstract

Accurate assessment of renal function is of great clinical and scientific importance, as it is an important pharmacokinetic covariate of pivotal drugs. The iohexol clearance is nearly identical to the glomerular filtration rate, but its determination usually requires an intravenous injection and therefore bears intrinsic risks. This motivates to showcase an *“en passant”* approach to quantification of renal function without additional risk or blood sampling beyond routine care using real-world data. We enrolled 37 intensive care patients who received high doses of iohexol for computed tomography imaging, and quantified series of iohexol plasma concentrations by high-performance liquid chromatography (HPLC-UV). Iohexol clearance was derived by both log-linear regression and nonlinear least squares fitting and compared to glomerular filtration rate estimated by the CKD-EPI-2021 formulas. Nonlinear fitting not only turned out to be more accurate but also more robust in handling the irregularly timed data points. Concordance of iohexol clearance against estimations based on both creatinine and cystatin C showed a slightly higher bias (−3.44 mL/min/1.73 m^2^) compared to estimations based on creatinine alone (−0.76 mL/min/1.73 m^2^), but considerably narrower limits of agreement (±42.8 vs. 56 mL/min/1.73 m^2^) and higher Lin’s correlation (0.84 vs. 0.72). In summary, we have demonstrated the feasibility and performance of the *“en passant”* variant of the iohexol method in intensive care medicine and described a working protocol for its application in clinical practice and pharmacologic studies.

## 1 Introduction

Correct assessment of renal function is of great importance in intensive care medicine, given that critically ill patients frequently suffer from pre-existing chronic kidney disease (CKD) and undergo acute changes in kidney function due to their current critical condition, e.g., cardiogenic shock, sepsis, or hemorrhage. The glomerular filtration rate (GFR) is the most important pharmacokinetic covariate of pivotal drugs in intensive care, including antiinfectives, anticonvulsives, or anticoagulants (Udy et al., 2017). Dose adjustments based on imprecise markers can impair efficacy and safety of such drugs and even increase mortality (Camargo et al., 2019). Beyond being beneficial for patient care, a more accurate assessment of GFR could be worthwhile to enhance the data quality of pharmacometric research in intensive care medicine.

Routine estimation of glomerular filtration (eGFR) is typically based on endogenous markers such as creatinine and cystatin C. The quantification of serum creatinine is inexpensive, but endogenous creatinine production in the critically ill is highly variable (e.g., due to loss of muscle mass), rendering estimation of GFR on its basis inaccurate and slow to respond to fluctuations. While cystatin C is a more precise marker at the population level (Inker et al., 2021), it also provides only an approximation of the true GFR (Kirwan et al., 2013), being influenced by co-morbidities such as thyroid disease, inflammation, or diabetes (Stevens et al., 2009). Also, it is more costly and therefore less commonly used.

Using a defined dose of an exogenous marker eliminates most of the imprecision inherent to endogenous markers. The iohexol method is currently one of the preferred procedures for accurate measurement of GFR (mGFR) (Delanaye et al., 2016). Iohexol is a non-ionized contrast agent with ideal characteristics for GFR determination, with low protein binding (Mützel et al., 1980) and elimination only by glomerular filtration, without tubular secretion or reabsorption (Olsson et al., 1983). Protocols for iohexol-based measurements of GFR have previously been described and thoroughly investigated (Jacobsson, 1983; Brown and O’Reilly, 1991; Erley et al., 2001; Schwartz et al., 2006; Delanaye et al., 2018). Such protocols typically use a very low dose iohexol (e.g. 5 g) and apply multiple-, four-, two-, and even one-time sampling. While iohexol is considered rather safe with a very low incidence of complications (Gaspari et al., 2018), there is a residual potential for nephrotoxicity and allergic reactions. It is, however, frequently applied in critically ill patients for radiographic studies, where the diagnostic benefit clearly outweighs the small risk.

In the present study, we investigated the feasibility of a pragmatic “*en passant*” application of the iohexol mGFR method to residual blood specimens from intensive care patients who received high dose iohexol contrast-enhanced computed tomography (CT) imaging as part of regular treatment. The aim was to establish a standardized protocol that can be used in clinical and pharmacological studies.

## 2 Materials and methods

### 2.1 Study design

This prospective observational feasibility study was performed at the surgical intensive care unit (ICU) of the University Hospital Regensburg, Germany, between February and June 2023. The study was registered at the German Clinical Trials Register (DRKS00031449) and was approved by the Institutional Review Board of the University of Regensburg (23-3202_1-101). Adult patients who received iohexol (Accupaque™ 755 mg/mL) for contrast-enhanced CT imaging during their intensive care treatment and had no extrarenal elimination of iohexol (e.g., by dialysis) were eligible for inclusion. In case of repeated iohexol administrations, patients were allowed to participate more than once. Patient characteristics as well as timing and dosing of iohexol injections 3 days before and after the day of inclusion were identified from the patients’ medical records. Residual samples after arterial or venous blood gas analyses in heparinized test tubes for up to 96 h after iohexol administration were collected. These whole blood samples were centrifuged at 3,000 g for 10 min and approximately 300 µL plasma specimens were obtained. Samples were stored at −20°C, and subjected to quantitative analysis within 3 months.

### 2.2 Bioanalytical method

Plasma iohexol was quantified using a Prominence LC20 modular HPLC system equipped with an SPD-M30A PDA detector (set to 245 nm) and LabSolution software (Shimadzu, Duisburg, Germany). The autosampler was kept at 6°C, the column oven at 40°C. Separation was performed using a CORTECS T3 2.7 µ 100 mm × 3 mm analytical column (Waters, Eschborn, Germany) preceded by a guard column protection system (Nucleoshell RP18 2.7 µ 4 mm × 3 mm, Macherey-Nagel, Düren, Germany). The mobile phase consisted of 0.1 M sodium phosphate buffer/acetonitrile 96:4 (v/v), final pH 3.0. Plasma sample treatment included deproteinization of 50 µL plasma with 200 µL 7% perchloric acid, incubation for 15 min at 4°C and centrifugation (3 min/10,500 g) to separate the precipitated proteins. An aliquot of 1 µL was injected into the HPLC. The retention time for iohexol was 3.3 min with a flow rate of 0.4 mL/min. Linearity in spiked plasma of healthy volunteers was given from 5 to 5,600 mg/L (R > 0.999). Based on in-process quality controls (concentrations of 2000/100/5 mg/L) the intra- and inter-assay imprecision of the determination of iohexol in plasma was < 5% (coefficient of variation), the relative error in accuracy was < 5%. Plasma creatinine and cystatin C were measured on the Cobas^®^ 8000 standard clinical chemistry analyzer modules C702 and C502 (Roche Diagnostics, Mannheim, Germany) with a colorimetric - kinetic Jaffee reaction and a particle enhanced turbidimetric assay, respectively.

### 2.3 Calculation of GFR

After single intravenous injection, iohexol distributes in the extracellular space within the first 2 h, after which the concentration-time curve in plasma can be described as negative mono-exponential function (Bröchner-Mortensen, 1972; Schwartz et al., 2006): *C* = *C*_0_ *e* ^−*kt*^. The parameters of this simple one-compartment model can be estimated by a fitting procedure. Both the slope-intercept (SI) and the nonlinear least squares (NLLS) optimization methods were applied in the present study.

**SI method:** After log-transformation of the concentration data, the intercept ln(*C*_0_) and slope *k* of a linear fit are determined by simple unweighted linear regression (Sapirstein et al., 1955). The volume of distribution is then given by $V_{d}=\frac{D}{C_{0}}$ , with *D* as the known iohexol dose, and the iohexol clearance follows as *Cl* = *k* ⋅ *V*_*d*_.

**NLLS method:** Estimates of the model parameters (*C*_0_ and *κ*) are derived by solving the unweighted least squares optimization problem iteratively using the Levenberg-Marquardt algorithm (Marquardt, 1963) on the original (non-transformed) concentration data.

Whenever multiple injections of iohexol occurred, the predicted residual concentration-time curve from the previous injection was subtracted from the subsequent data points. To correct for the neglection of the distribution phase of iohexol (the first 2 h after injection), the formula by Jødal and Brøchner-Mortensen was applied as $mGFR=\frac{Cl}{1+0.0032{BSA}^{-1.3}Cl}$ (Jødal and Brøchner-Mortensen, 2009), which takes into account the individual’s estimated body surface area (BSA). This yields the *measured* GFR (mGFR). Values were then normalized to estimated BSA as determined by the formula by Du Bois and Du Bois (Du Bois and Du Bois, 1916). Furthermore, GFR was also *estimated* by the CKD-EPI-2021 formulas, taking into account patient characteristics (sex and age) and creatinine (Eq. 1), or patient characteristics, creatinine, and cystatin C (Eq. 2).

$${eGFR}_{\text{cr}}=142\cdot \min{\left(\frac{Scr}{\kappa },1\right)}^{\alpha }\cdot \max{\left(\frac{Scr}{\kappa },1\right)}^{-1.2}\cdot 0.9938^{\text{age}}\cdot \left(1.012\;\left[\text{if female}\right]\right)$$ (1)

where Scr is serum creatinine, *κ* is 0.7 for females and 0.9 males, *α* is −0.241 for females and −0.302 for males.

$$\begin{array}{c} {eGFR}_{\text{cr,cys}}=135\cdot \min{\left(\frac{Scr}{\kappa },1\right)}^{\alpha }\cdot \max{\left(\frac{Scr}{\kappa },1\right)}^{-0.544}\cdot \min{\left(\frac{Scys}{0.8},1\right)}^{-0.323}\cdot \max{\left(\frac{Scys}{0.8},1\right)}^{-0.778}\\ \cdot 0.9961^{\text{age}}\cdot \left(0.963\left[\text{if female}\right]\right) \end{array}$$ (2)

where *κ* is 0.7 for females and 0.9 males, *α* is −0.219 for females and −0.144 for males (Inker et al., 2021). Creatinine and cystatin C were measured on the day of iohexol administration.

### 2.4 Statistics

Patient data were recorded in LibreOffice Calc 7.3, then processed and analyzed in Python 3 with JupyterLab 4.0. The simple linear regression model was programmed using the scikit-learn 1.3 library. The nonlinear model and statistics were programmed using the scipy 1.11 library, and matplotlib 3.7 was used for visualizations. The bootstrap analysis was also coded in Python 3. Descriptive data are presented as number with percentage or median with min-max range. All statistics were calculated on an untransformed original scale, unless otherwise specified. The coefficient of determination and the root mean square error (RMSE) are used to describe the model fits. Lin’s concordance correlation coefficient and Bland-Altman analysis are used to describe the agreement of the different methods to determine GFR, where bias is defined as mean difference between two variables, and the limits of agreement (LOA) as 1.96 standard deviations.

## 3 Results

Data from 37 intensive care patients who underwent contrast-enhanced CT scans were collected in the present study. All 37 subjects were Caucasian. Ten out of these 37 subjects had received iohexol on multiple occasions and were therefore included more than once, resulting in a total of 48 concentration-time curves with 488 plasma specimens subjected to iohexol quantification. Due to the exclusive use of residual blood samples from routine patient care, the timing of the data points was variable.

### 3.1 Eligible data points for the calculation of iohexol clearance

**FIGURE 1:**
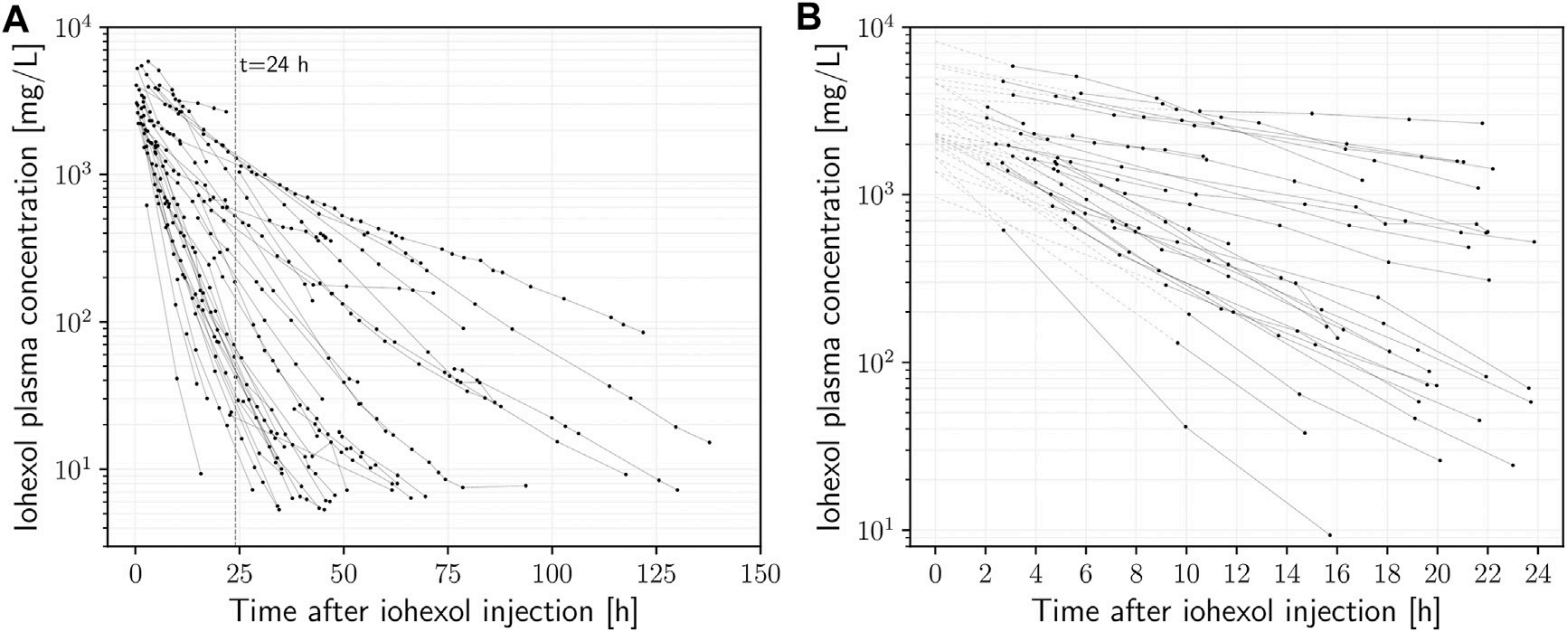
**(A)** Plasma iohexol semi-logarithmic concentration-time curves (n = 27) of intensive care patients receiving iohexol contrast-enhanced CT imaging. **(B)** Data points used for calculation of mGFR ( > 2 and < 24 h). Dotted lines depict the back-extrapolation to *t* = 0 based on the nonlinear least squares method (NLLS) fitted models’ estimates.

The onset of the mono-exponential elimination phase (i.e., the time at which the distribution phase of iohexol is completed) was determined by visual inspection on a semi-logarithmic scale. In several cases, distribution took place up to 2 h after iohexol injection. Inspection also showed that the concentration-time curves often began to exhibit fluctuations in slope after about 24–36 h. Therefore, to be consistent in the context of this proof of concept, only measurements between two and 24 h after the time of injection were used (140 out of the 488 plasma specimens) to estimate the model parameters. A full overview of all attained samples and their timings can be seen in Supplementary Figure S1. The initial fits revealed that the residual error variability considerably increases with increasing delay between injection of iohexol and the first concentration measurement (Supplementary Figures S2, S3). On that basis, 12 h after injection was used as the upper limit for the first time point. The delay of the first measurement was longer than that in four cases, which were therefore excluded from further analysis. This led to a final number of 27 investigated subjects and a total of n = 32 eligible concentration-time curves for calculation of the GFR, based on a total of 140 samples (median 5 (range 2–13) concentrations per curve). Table 1 shows the characteristics of the final patient cohort. Iohexol doses ranged from 52.8 to 181.2 g (median 75.5 g). The concentration-time curves of all recruited subjects are given in Figure 1A, while Figure 1B depicts the curves used as basis for calculation of the mGFR after application of the above mentioned criteria.

**TABLE 1:** Demographic and other characteristics of the subjects included in the final analysis (n = 27). Primary medical specialty is based on the main reason for ICU admission.

Demographics	n (%)
Male/female	18/9 (66.7/33.3)
	median (range)
Age [yr]	63 (35–90)
Weight [kg]	80 (50–120)
Height [cm]	175 (160–190)
BSA [m^2^]	1.9 (1.51–2.38)
Other characteristics	median (range)
ICU length of stay [d]	6 (1–52)
Days on a ventilator [d]	5 (1–42)
Days on Noradrenaline ( > 0.5 mg/h) [d]	4 (1–32)
SOFA score on the day of inclusion [-]	4 (0–13)
Admission creatinine [mg/dL]	1.01 (0.56–2.42)
	n (%)
Known chronic kidney disease	4 (14.8)
Acute kidney failure	6 (22.2)
Sepsis	4 (14.8)
ICU mortality	5 (18.5)
Medical specialty	n (%)
Traumatology	10 (37)
General surgery	10 (37)
Neurosurgery	3 (11.2)
Vascular surgery	2 (7.4)
Internal medicine	2 (7.4)

### 3.2 Fitting metrics

**FIGURE 2:**
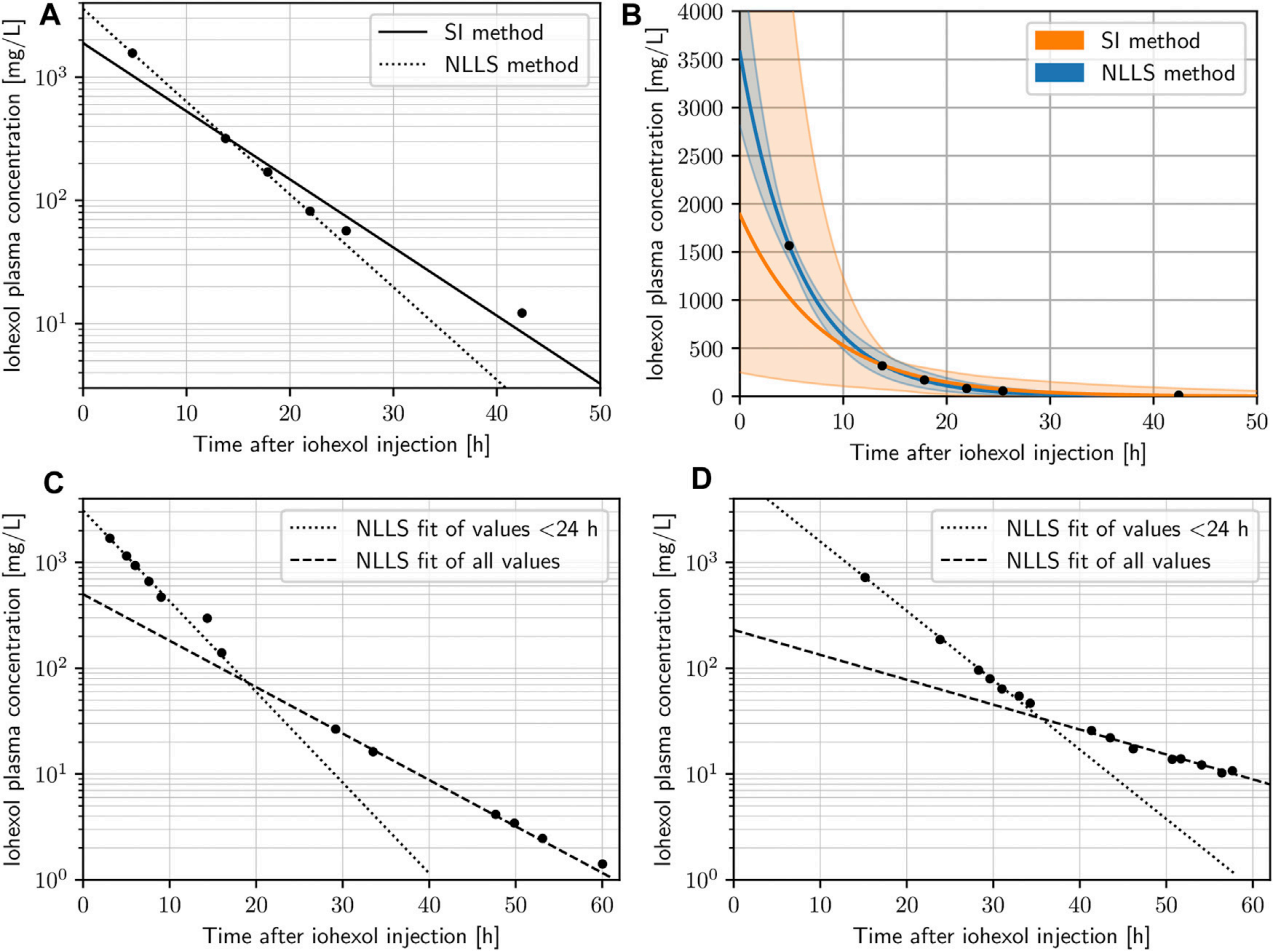
**(A)** Concentration-time data points of a given subject with both nonlinear least squares (NLLS) method (dotted) and the slope-intercept (SI) method (dashed) fits on a semi-logarithmic scale. **(B)** Concentration-time data points of a given subject with both NLLS method (blue) and SI method (orange) fits on the original scale, highlighting the level of uncertainty of predictions (color bands) based on a bootstrapping simulation of 1,000 iterations for each data point adding a normally distributed random error. **(C,D)** Concentration-time data points of two subjects on the semi-logarithmic scale highlighting changes in the subjects physiology with an NLLS fit using the values < 24 only (dotted) and an NLLS fit using all values (dashed).

All n = 32 curves included in the final analysis displayed adequate fits by visual inspection on the original and semi-logarithmic scales. The pooled standardized residuals for both NLLS and SI method fits can be seen in Supplementary Figures S4, S5. NLLS fits for curves with more than two plasma concentrations yielded a median *R* ^2^ of 0.995 (range 0.956–0.999) with a median RMSE of 27.48 mg/L (range 2.32–304.1 mg/L), and the SI method resulted in a median *R* ^2^ of 0.991 (range 0.944–0.999) with a median RMSE of 38.72 mg/L (range 3.85–416.27 mg/L). In two instances only two plasma specimens were available (*R* ^2^ = 1 and RMSE = 0). Based on the metrics and visual inspection, NLLS was deemed the more adequate and reliable method in the extrapolation of the curves backwards to *t* = 0 and thus more robust to determine the PK parameters, particularly *C*_0_. Figure 2 shows examples of concentration-time curves from the dataset, highlighting the superiority of NLLS in determining *C*_0_, as the SI method implicitly puts more weight on the late values. To illustrate this effect even more clearly, we resampled the data points in a bootstrapping simulation with 1,000 iterations including an additional normally distributed random error term with mean zero and a standard deviation of 50 mg/L, and plotted the variability of the predictions as a color band (Figure 2B). The superiority of NLLS can be clearly seen, with much larger uncertainty in the extrapolation of the data back to *t* = 0, when the SI method is used. Analyses given in the following sections are based on fits that apply the NLLS method.

### 3.3 Estimated and measured GFR

**FIGURE 3:**
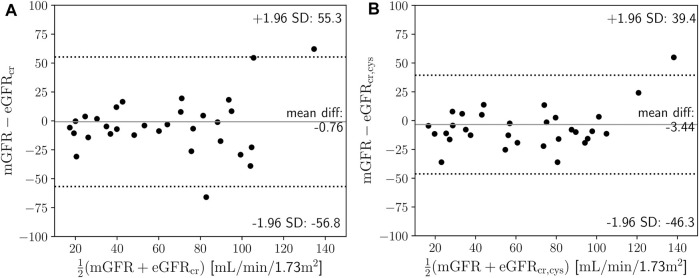
**(A)** Mean difference plot of measured glomerular filtration rate (mGFR) based on iohexol clearance against creatinine-based estimated glomerular filtration rate (eGFR_cr_) (Eq. 1). **(B)** Mean difference plot of mGFR based on iohexol clearance against creatinine- and cystatin C-based eGFR_cr,cys_ (Eq. 2). SD is standard deviation.

Estimations of the GFR with the CKD-EPI-2021 formulas resulted in: median 66.7 mL/min/1.73 m^2^ (range 20.2–123.6 mL/min/1.73 m^2^) for eGFR_cr_, and median 70.3 mL/min/1.73 m^2^ (range 18.9–131.6 mL/min/1.73 m^2^) for eGFR_cr,cys_. Measured GFR by means of the iohexol clearance using NLLS fitting resulted in a median of 62.6 mL/min/1.73 m^2^ (range 4.7–203.9 mL/min/1.73 m^2^), and median volume of distribution of 32.9 L (range 14.6–67 L). The distributions of the GFR and *V*_*d*_ are shown in Supplementary Figures S6, S7. Bland-Altman analysis yielded a bias (±LOA) of −0.76 ± 56 mL/min/1.73 m^2^ for mGFR against eGFR_cr_ (Figure 3A), and of −3.44 ± 42.8 mL/min/1.73 m^2^ for mGFR against eGFR_cr,cys_ (Figure 3B), with a Lin’s concordance correlation coefficient of 0.72 and 0.83, respectively.

### 3.4 Examples of clinically relevant findings

On inspection of the concentration-time curves (Figure 1A) and the resulting GFRs, we were able to easily identify multiple cases in which considerable deviations of the eGFR from the mGFR occurred to an extent that could also be clinically relevant. For example, one subject showed an acute deterioration in renal function, reflected by a decrease in mGFR between multiple measurements of 88% within 48 h (from 31.46 to 3.77 mL/min/1.73m^2^), while creatinine-based eGFR showed only a drop of 33.9% (from 54.38 to 35.92 mL/min/1.73m^2^). The eGFR revealed the severity of the acute renal impairment only after an additional delay of approximately 2 days, dropping by 60% from 35.92 on day two to 14.31 mL/min/1.73m^2^ on day four (Supplementary Table S8). As another example, a given subject showed signs of augmented renal clearance with very rapid elimination of iohexol on visual inspection of the data. The mGFR was 175.2 mL/min/1.73m^2^ while creatinine-based estimation with 114 mL/min/1.73m^2^ seemed to considerably underestimate the GFR.

## 4 Discussion

To our knowledge, this study is the first to demonstrate the feasibility of *“en passant”* quantification of the glomerular filtration rate based on the exploitation of coincidental administration of high-dose iohexol for computed tomography imaging. We detail the procedural requirements for the implementation of additive mGFR monitoring in intensive care, that could be particularly useful for clinical and pharmacometric studies. Estimated from retrospective data in our surgical ICU, a patient receives a contrast-enhanced CT scan on average every 7 days. It therefore seems quite realistic that our technique can be applied regularly to this cohort - at least once during a patient’s course of treatment in intensive care. The key advantages of our “*en passant*” iohexol method are twofold: Firstly, it allows the quantification of renal function utilizing exclusively residual blood samples obtained during routine clinical care, and secondly, unlike alternative methods, it does not require urine collection (e.g., creatinine urine clearance) and spares patients from the additional risks associated with intravenous injection of exogenous markers. Interestingly, opportunistic CT urography-based approaches to measure the GFR have also recently been described, showing good agreement with iohexol clearance-based mGFR (Stehlé et al., 2023), again highlighting the value of maximizing the use of data from routine patient care. CT-based protocols typically involve CT-urography exams using iopromide or iomeprol as the contrast agent, and may have advantages over other techniques. E.g., the ability to identify asymmetric renal disease (Hackstein et al., 2001; Hackstein et al., 2004; Yuan et al., 2016; You et al., 2018; Jeong et al., 2021; Stehlé et al., 2023). The widely used creatinine-based method for estimating GFR, however, is known to be quite inaccurate. It has recently been shown that it tends to overestimate GFR in intensive care patients (Sangla et al., 2020), particularly with longer length of stay in the ICU which is related to continuous loss of muscle mass (Haines et al., 2023). Also, the eGFR has been shown to underestimate GFR in the presence of augmented renal clearance (Bilbao-Meseguer et al., 2018; Collet et al., 2021), which aligns well with our findings presented in Section 3.4, while creatinine clearance can lead to significant underestimation when compared to the iohexol clearance as a gold standard. (Collet et al., 2021). On the other hand, it is known that iohexol clearance aligns closely with the actual GFR and is therefore a more reliable marker. The resulting mGFR values from our “*en passant*” approach showed agreement with eGFR_cy,cys_ comparable to recent data for intensive care patients under ideal conditions, which described a bias of −11 ± 51.5 mL/min/1.73 m^2^ (Sangla et al., 2020), while our data showed −3.44 ± 42.8 mL/min/1.73 m^2^.

The classic iohexol method has long been established and validated (Erley et al., 2001; Schwartz et al., 2006; Delanaye et al., 2016; Carrara and Gaspari, 2018; Sangla et al., 2020; Dhont et al., 2022). In conventional protocols, a very low dose of iohexol (e.g. 5 g) is used and sampling is carried out according to a strict schedule. In contrast, we demonstrated the feasibility of calculating GFR by iohexol clearance after *high doses* and effectively *randomly timed* concentration measurements. The impact of the timing of concentration measurements on the accuracy of mGFR calculation is a topic of intense discussion (Stolz et al., 2010; Delanaye et al., 2016; Baklouti et al., 2021) and both extensive and limited sampling approaches have been described (Sarem et al., 2015). A crucial step is hereby the extrapolation of the concentration-time curve back to the time of iohexol injection *t* = 0 in order to get *C*_0_ for subsequent calculation of $V_{d}=\frac{D}{C_{0}}$ and Cl_ihx_ = *κ* ⋅ *V*_*d*_. It is important that the first concentration measurement is taken as early as possible after completion of the distribution phase (after approximately 2 h), as an increasing delay between the injection of iohexol and the first concentration data introduces additional variability in the determination of the PK parameters (Supplementary Figures S2, S3). The choice of fitting algorithm for estimating the model parameters can also have a considerable impact on the accuracy of the results, which we believe is generally not given enough attention. The SI method usually applies simple linear regression and minimizes the sum of the squared residuals of log-transformed concentrations to fit the observations. Therefore, the late values are implicitly weighted more heavily. NLLS, on the other hand, solves the least squares problem on the original scale, and therefore fits the PK parameters more accurately to the “real” (untransformed) observations, as was shown under ideal conditions in the recent study by Pottel et al. (2021). This effect is further demonstrated in Figures 2A–D based on cases from our dataset. It is apparent that nonlinear least squares fitting gives more appropriate weight to the late concentrations, based on their absolute values. However, the effects on the total area under the curve (AUC) seem relatively small. The extrapolation of the data back to the ordinate (*t* = 0), on the other hand, is considerably more volatile and can lead to distorted PK parameters, as seen in the bootstrapping analysis in Figure 2B.

It is also important to recognize that the described models of renal elimination work under the assumption of constant physiology during the investigated time-frame. This assumption needs to be considered carefully, particularly in intensive care patients where acute changes frequently occur. When reconstructing a concentration-time curve by a limited number of data points, such fluctuations will create difficulties in fitting the model to the data, and the likelihood to encounter fluctuations in patient physiology (most importantly renal function) increases with the length of the data acquisition. In our dataset, the slopes of the concentration-time curves are relatively constant during the first 24 h after injection, but then a considerable number start to experience fluctuations (Figure 1). This is best illustrated by the examples in Figures 2C, D, which show almost perfect log-linear behavior up to a point between 24 and 36 h, where their slope changes (e.g. deterioration of renal function). Notably, this also demonstrates for early detection of acute renal failure, when our method is applied.

Some limitations of the presented study should be mentioned. As a proof-of-concept, in line with the opportunistic “*en passant*” character of the method, sampling followed clinical requirements only, and not an optimized schedule. Particularly in the early phase (two to 12 h), data points were scarce or completely lacking in some patients (Supplementary Figure S1). The coefficient of determination and the root mean square error were used as goodness of fits metrics. While they are useful measures to identify very inappropriate fits, they are not able to reliably determine goodness of fit of nonlinear models. However, our goal was not to research and validate different fitting methods, but rather determine an adequate strategy for an “*en passant*” protocol. Also, as the only direct validation of our mGFR values, we compared to the eGFR and not a reference standard. However, mGFR with iohexol itself is accepted as the current gold standard when strict sampling schemes are followed. Still, larger studies involving a different, more reliable method of cross-checking renal function than the CKD-EPI-2021 for comparison are needed. In particular, the urinary clearance of iohexol seems to be an excellent candidate, with, e.g., inulin or ^51^Cr-EDTA clearance being reasonable alternatives. Moreover, the purpose of this study was to demonstrate the feasibility of the “*en passant*” approach using pragmatic real-world data, and not to validate (or invalidate) estimates of GFR. The pitfalls and imprecision of eGFR based on creatinine and also cystatin C in critically ill patients are well known (Stevens et al., 2009; Kirwan et al., 2013; Haines et al., 2023).

In conclusion, the “en passant” iohexol method is a versatile tool that could in the future be used to improve targeted therapies as well as pharmacokinetic research. E.g., to reduce unexplained variability in pharmacokinetic models, to assess protein binding of drugs (as renal drug clearance divided by iohexol clearance), or to determine the volume of distribution of iohexol within a PK study as a reference to improve our understanding the distribution of other drugs.

Based on our findings and previous guidelines for the iohexol method (Carrara and Gaspari, 2018), the following recommendations to the “*en passant*” approach seem reasonable: 1) The first measurement should be close to the beginning of the elimination phase at 2 h but no earlier, 2) limit the length of the interval for which the GFR is to be calculated to minimize the risk of physiologic changes occurring during the investigation, 3) NLLS should be used for curve fitting rather than the SI method, and 4) data should be visually inspected to identify acute changes in renal function. Only the first section of the concentration-time curve, i.e., before any significant fluctuations occur, should then be used for calculation of mGFR. The calculated GFR will be valid for that interval.

## Data Availability

The raw data supporting the conclusion of this article will be made available by the authors, without undue reservation.
