# Supplementary material for: Determination of glomerular filtration rate “en passant” after high doses of iohexol for computed tomography in intensive care medicine—a proof of concept
Source: Front Pharmacol. 2024 Feb 1;15:1346343. doi: 10.3389/fphar.2024.1346343 (PMC10867190; doi:10.3389/fphar.2024.1346343)
Supplement: Supplementary file 1 [file DataSheet1.pdf]

Supplementary Material

1 SUPPLEMENTARY FIGURES

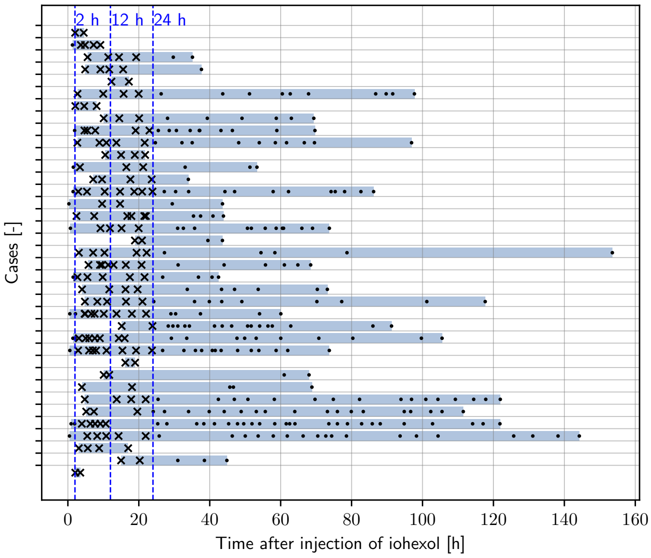

Figure S1. All $n = 44$ cases are shown with the timing of all available blood samples. Samples used in the final analysis are shown as "x", samples which were excluded are shown as ".". Blue lines at 12 and 24 h depict the cut-offs for inclusion described in the methods section.

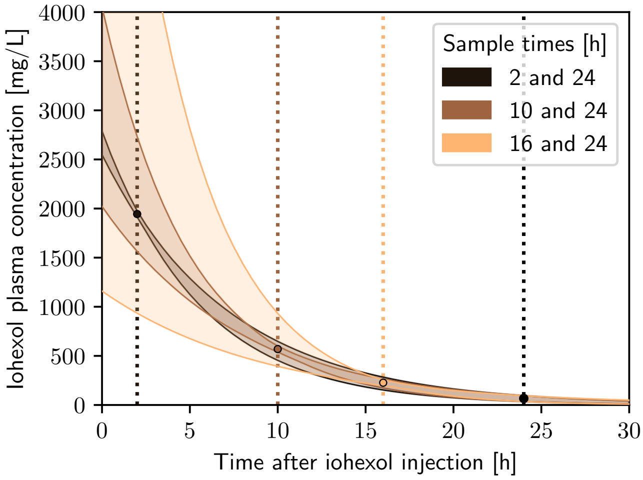

Figure S2. Concentration-time curves are shown based on a bootstrapping simulation with 1000 iterations for each data point, adding a normally distributed random error. Three curves are shown which represent nonlinear least squares fits based on two observations: at 2 and 24 h, 10 and 24h, and 16 and 24 h. The uncertainty of the fit is highlighted as color band, which shows the significant increase in uncertainty when samples are late and close together.

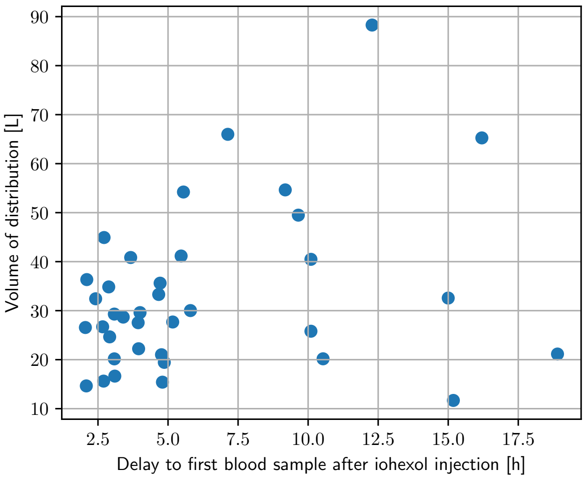

Figure S3. The calculated volume of distribution V_d based on the nonlinear least squares fit is shown against the time of the first blood sample for each case, demonstrating the increase in variability in V_d with increasing delay between time of injection of iohexol and the first blood draw.

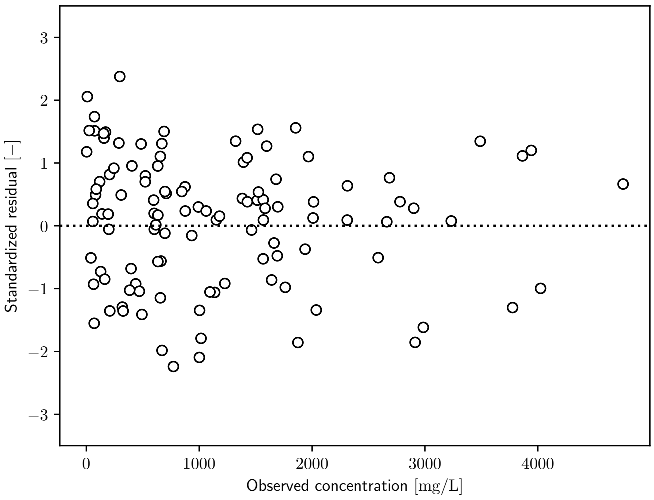

Figure S4. Pooled standardized residuals (observed-predicted) of iohexol concentrations based on the nonlinear least squares method fits. Only data and subjects who were included in the final analysis are shown (only data points between two and 24 hours).

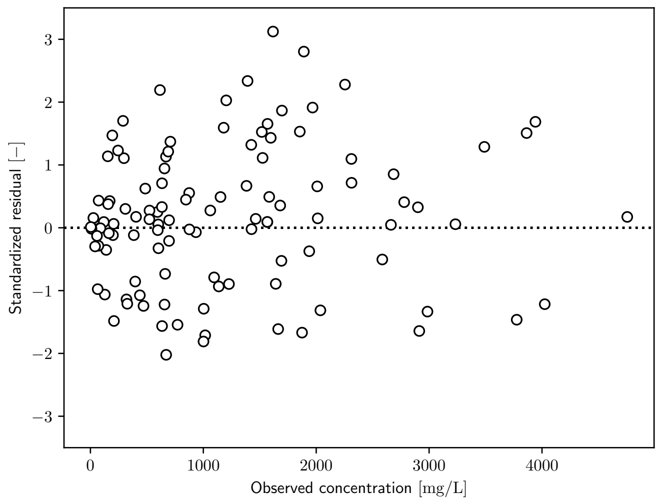

Figure S5. Pooled standardized residuals (observed-predicted) of iohexol concentrations based on the slope-intercept method fits. Only data and subjects who were included in the final analysis are shown (only data points between two and 24 hours).

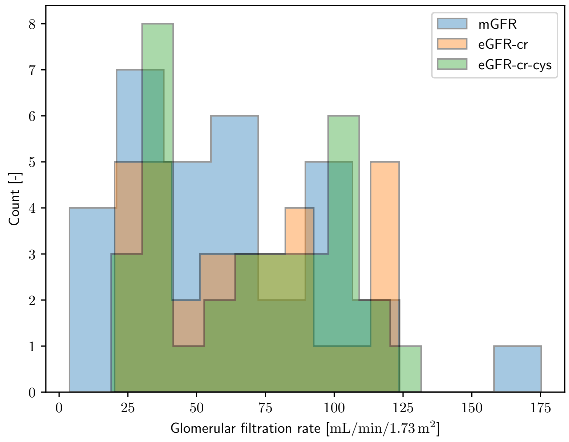

Figure S6. A combined histogram of the calculated GFR values: measured glomerular filtration rate (mGFR), creatinine-based estimated glomerular filtration rate ($eGFR_{cr}$), creatinine- and cystatin C-based estimated glomerular filtration rate ($eGFR_{cr,cys}$).

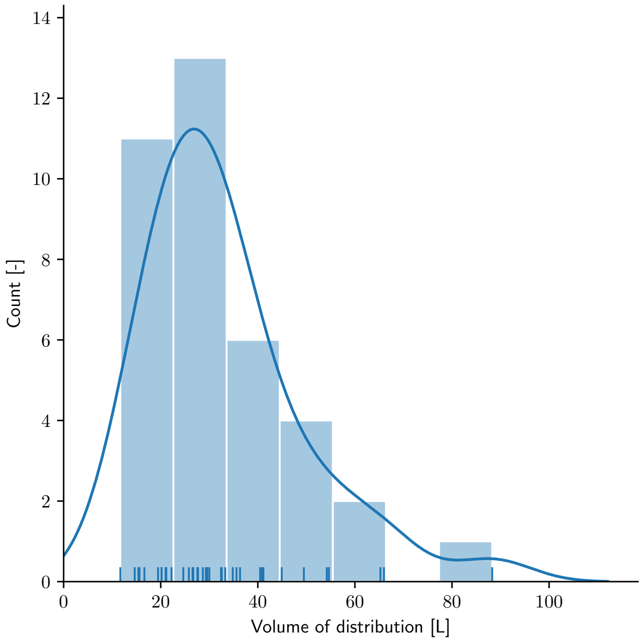

Figure S7. A histogram of the calculated volumes of distribution based on nonlinear least squares fits.

2 SUPPLEMENTARY TABLES

Table S8. Creatinine, cystatin C, measured glomerular filtration rate (mGFR), and estimated glomerular filtration rate (eGFR) by creatinine-based CKD-EPI-2021 formula of a given patient from the study's dataset with signs of acute renal failure.

Days after enrollment	Creatinine [mg/dL]	Cystatin C [mg/dL]	mGFR [mL/min/1.73m^2]	eGFR$_{cr}$ [mL/min/1.73m^2]
0	1.43	1.02	31.46	54.38
2	2.02	1.51	3.77	35.92
4	4.35	-	-	14.31
